# Fabrication of Nanoscale Oxide Textured Surfaces on Polymers

**DOI:** 10.3390/polym13132209

**Published:** 2021-07-03

**Authors:** Barun K. Barick, Neta Shomrat, Uri Green, Zohar Katzman, Tamar Segal-Peretz

**Affiliations:** 1 Department of Chemical Engineering, Technion, Haifa 3200003, Israel; barun.barick@weizmann.ac.il (B.K.B.); sneta@trdf.technion.ac.il (N.S.); 2 Shamir Optical Industry Ltd., Kibbutz Shamir, Upper Galilee 1213500, Israel; urig@shamir.co.il (U.G.); zkatzman@shamir.co.il (Z.K.)

**Keywords:** block copolymer, solvent vapor annealing, sequential infiltration synthesis, nanotexture, surface passivation

## Abstract

Nanoscale textured surfaces play an important role in creating antibacterial surfaces, broadband anti-reflective properties, and super-hydrophobicity in many technological systems. Creating nanoscale oxide textures on polymer substrates for applications such as ophthalmic lenses and flexible electronics imposes additional challenges over conventional nanofabrication processes since polymer substrates are typically temperature-sensitive and chemically reactive. In this study, we investigated and developed nanofabrication methodologies to create highly ordered oxide nanostructures on top of polymer substrates without any lithography process. We developed suitable block copolymer self-assembly, sequential infiltration synthesis (SIS), and reactive ion etching (RIE) for processes on polymer substrates. Importantly, to prevent damage to the temperature-sensitive polymer and polymer/oxide interface, we developed the process to be entirely performed at low temperatures, that is, below 80 °C, using a combination of UV crosslinking, solvent annealing, and modified SIS and RIE processes. In addition, we developed a substrate passivation process to overcome reactivity between the polymer substrate and the SIS precursors as well as a high precision RIE process to enable deep etching into the thermally insulated substrate. These methodologies widen the possibilities of nanofabrication on polymers.

## 1. Introduction

Oxide nanoscale structures play a central role in optical, electrical, and biomedical nanotechnological devices and sensors due to their tunable optoelectronic properties, high surface-to-volume ratio, and good stability [1,2]. In particular, surface texturing with high-aspect-ratio oxide nanostructures significantly enhances surface-based properties in sensors [3,4], antibacterial surfaces [5,6], hydrophobic surfaces [7], and optical lenses [8,9]. Oxide surface texturing has been demonstrated by several nanostructure formations and patterning techniques, including photolithography [10], nanoimprint lithography [11,12], colloid assembly [13,14], and mesoporous silica layers [15]. A high-aspect-ratio oxide nanostructure can enhance properties such as light transmission and surface hydrophobicity as well as act as an antireflective layer due to the low effective refractive index of the metal oxide/air sub-wavelength nanostructure [11]. If the nanostructure is designed to create an effective refractive index gradient, it can exhibit wide angular broadband anti-reflective behavior, such as moth eyes exhibit in nature and as has been mimicked by several man-made approaches [12,16,17,18,19,20,21,22,23].

Recent work has demonstrated oxide nanoscale structures on silicon substrates [23]. However, surface texturing of polymer substrates imposes new challenges compared to inorganic substrates due to additional constraints on the processing temperatures as well as polymer reactivity. With the growing demand for lightweight and cost-effective devices, there is a need for new nanofabrication processes for polymeric substrates [24].

In the past few decades, block copolymer (BCP) self-assembly has emerged as an excellent nanofabrication technique for simple and scalable nanoscale patterning [25,26,27,28,29]. BCP self-assembly yields highly ordered and uniform nanostructures with periodicity ranging between 10 and 100 nm [29]. BCP patterning is typically performed by casting a thin BCP layer on the substrate and annealing it to induce phase separation [28]. The BCP pattern can then be transferred to the underlying inorganic layer, resulting in a textured surface that can enhance hydrophobicity or anti-reflective properties [30,31]. However, pattern transfer of the BCP layer into high aspect ratio structures can impose a challenge due to the low etch contrast between typical BCP organic-organic domains.

Recently, sequential infiltration synthesis (SIS) has emerged as a novel technique for selectively growing metal oxides within the polar domain of block copolymers, enabling a high etch contrast between the blocks and efficient pattern transfer to form high-aspect-ratio structures [23,32,33,34,35,36]. SIS is based on atomic layer deposition (ALD) chemistry, where high precursor partial pressures and long exposure times result in precursors’ sorption and diffusion within the polymer [37]. The favorable interaction between the organometallic precursors and the polar domains of BCP can lead to the selective growth of metal oxides such as AlO_x_ [35], ZnO [38], TiO_x_ [39], and SnO_x_ [40] in only one domain of the block copolymers [25,32,33,36]. Following the SIS process, the BCP film can be removed, resulting in a metal oxide nanostructure templated by the BCP morphology with high fidelity [25]. SIS has been successfully implemented into nanofabrication processes, such as semiconductor patterning [25,38], porous membrane fabrication [32,41,42], and anti-reflective layers [23,43].

Until now, SIS-based metal oxide nanostructures were fabricated onto silicon wafers or glass substrates, which are chemically inert and can withstand high-temperature processes. On the other hand, polymer substrates, such as diglycol, thiosulfonate, polycarbonate, and others, are not only sensitive to temperature but also can interact with the organometallic precursors used in the SIS process. Combining BCP self-assembly and SIS could offer a simple, scalable, and efficient way to fabricate high-aspect-ratio nanoscale oxide textures over a polymeric substrate.

In this study, we aim to expand SIS processes to polymer substrates and to enable nanoscale texturing of oxides onto polymers. Working with polymeric substrates poses a two-front challenge. First, there are competing temperature needs for optimal BCP assembly, SIS, and etching processing vs. the polymer substrate Tg and the polymer/oxide layer thermal expansion properties. Second, unlike silicon or glass inert substrates, polymers are potentially chemically reactive through outgassing or terminal edge moieties. The approach and methodologies developed in this work mitigate these challenges by using a combination of polymer substrate passivation processes to prevent undesired interactions between SIS precursors and the polymer substrates, room-temperature BCP assembly processes, and composite SIS patterning that overcomes pattern transfer challenges.

## 2. Experimental Details

### 2.1. Materials

BCP: polystyrene-*block*-poly(methyl methacrylate) (PS-*b*-PMMA) (Mn: 140k-*b*-65k, PDI = 1.16), (Mn:46.1k-*b*-21k, PDI = 1.09) (Mn: 68k-*b*-33.5k, PDI = 1.08), metalorganic precursors (trimethyl aluminum (TMA), diethyl zinc (DEZ)) and solvents (toluene, tetrahydrofuran, chloroform, etc.) were purchased from Polymer Source, STREM chemicals, and Fisher Scientific, respectively, and used as received.

### 2.2. Substrate Preparation

Discs of diglycol (MR-8^TM^), thiosulfonate (CR-39^TM^), and polycarbonate with a diameter and thickness of 20 mm and 2 mm, respectively, low resistivity (<0.005 Ω.cm) 4′′ Si (100) substrates, and glass slides were coated with 350 nm of SiO_2_ using chemical vapor deposition (MC380X box coater, Satisloh, Germantown, WI, USA) at Shamir Optics, Israel. SiO_2_/Si and SiO_2_/glass substrates were used as the reference standards. The surfaces of all the SiO_2_-coated substrates were cleaned in O_2_ plasma for 5 min prior to further processing.

### 2.3. BCP Templates

A thin layer of poly(styrene-*r*-methyl methacrylate-*r*-glycidyl methacrylate) (P(S-*r*-MMA-*r*-GMA)) containing ~4 mol% of glycidyl methacrylate with styrene mole fractions of 77% (PG-4 76%PS) was used for tuning the surface interactions of PS-*b*-PMMA with SiO_2_ substrate by spin-coating 0.3 wt. % of it in toluene. P(S-*r*-MMA-*r*-GMA) was synthesized by reversible addition fragmentation chain transfer polymerization with Azobisisobutyronitrile (AIBN), which is a thermal and photoinitiator. UV crosslinking was performed using Spectrolinker™ UV Crosslinkers (XL-1000 model) at a wavelength of 254 nm in N_2_ atmosphere. A layer of PMMA cylinder-forming PS-*b*-PMMA was fabricated by spin-coating 2 wt. % solution of PS-*b*-PMMA in toluene. The BCP layer was solvent vapor annealed (SVA) by exposing it to saturated vapors of tetrahydrofuran in a closed chamber containing ~100 mL of solvent at room temperature for durations ranging from 5 to 30 min. After exposure, the SVA was immediately quenched by taking it out of the annealing chamber, and the film was kept inside a fume hood for ~4 h to complete the evaporation of trapped THF vapors.

### 2.4. Sequential Infiltration Synthesis

AlO_x_ and ZnO SIS were performed in a Savannah S100 ALD system (Veeco, New York, NY, USA). BCP films were loaded into the ALD reactor at 80 °C. To achieve thermal equilibrium and remove excess moisture, the samples were subjected to 20 sccm of N_2_ flow at 0.3 Torr for at least 30 min. The SIS process was carried out as follows: upon TMA pulse, the chamber was closed in a static mode for 300 s, enabling precursor diffusion into the polymer film (exposure). The exposure step was followed by an N_2_ purge step for 350 s to remove excess reactants. A similar exposure/purge process was used for water. The full AlO_x_ SIS cycle, TMA/purge/H2O/purge = 300s/350s/300s/350s, was repeated several times. In a similar approach, AlO_x_/ZnO composite cylinders were fabricated by 2 cycles of AlO_x_ SIS, followed by 6 cycles of ZnO SIS (DEZ/purge/H_2_O/purge) using the same sequence as AlO_x_ SIS. After completion of the SIS process, the BCP template was removed by O_2_ plasma etching (50 W, 0.4 mbar for 10–20 min).

### 2.5. Passivation Process

The SiO_2_/polymer substrate was positioned over three adjacently placed Teflon discs to avoid the direct thermal contact of the polymer substrate with the ALD chamber. The passivation was performed by 100 cycles of Al_2_O_3_ ALD cycles using the following sequence: TMA exposure/purge/H_2_O exposure/purge for 10s /10s/10s/10s, respectively, at 80 °C.

### 2.6. Reactive Ion Etching

Vertically aligned SiO_2_ nanorods were fabricated by inductively coupled plasma-reactive ion etching using a Plasma-Therm (Oxford Instruments, Model: 790) with a CHF_3_ (5 sccm, 12 mTorr, 97 W rf power) and CF_4_ + O_2_ (36 sccm of CF_4_, 2 sccm of O_2_, 40 mTorr, 175 W rf power) mixture for different durations at room temperature.

### 2.7. Characterizations

The morphology of the sample was studied by a high-resolution scanning electron microscope (Ultra-Plus HRSEM, Zeiss, Oberkochen, Germany) at 1 kV. SEM cross-sectional samples on a SiO_2_/Si substrate were prepared by cleaving the substrate along a crystallographic direction. SiO_2_/polymer lenses were coated with 3 nm of Pt using BAF (Leica BAF 060) and cleaved after dipping them inside liquid nitrogen. Cross-section images were recorded by tilting the sample by 70°.

## 3. Results and Discussion

### 3.1. Process Overview

**Figure 1:**
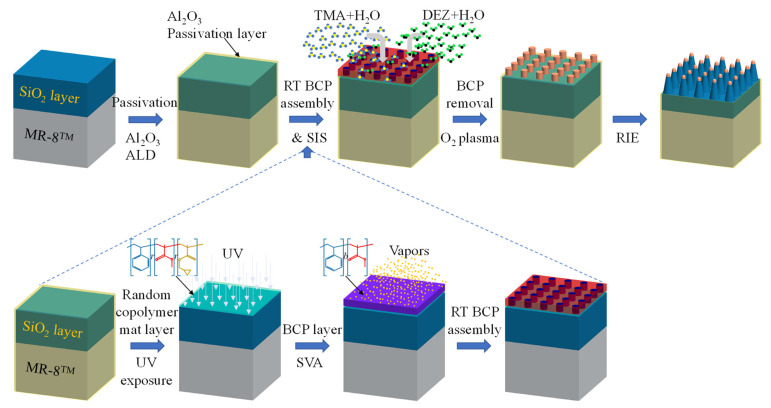
Schematic illustration of the developed processes. The process starts with SiO_2_/MR-8^TM^ polymeric substrate passivation using Al_2_O_3_ ALD, followed by room-temperature BCP assembly—random copolymer mat layer crosslinking using UV exposure and SVA of cylinder-forming PS-*b*-PMMA to promote self-assembly. Growth of AlO_x_ and ZnO within the BCP layer is performed using TMA/H_2_O and DEZ/H_2_O SIS processes, followed by polymer template removal. Finally, the pattern is transferred to the SiO_2_ layer with RIE.

The fabrication methodology and processes for the nanoscale texturing of oxide layers on polymer substrates are schematically illustrated in Figure 1. The process was designed to enable the desired interaction between the BCP layer and SIS precursors while hindering interactions between these precursors and the polymer substrate. MR-8^TM^ was chosen as the polymer substrate model system due to its wide use in ophthalmic lenses. Other polymer substrates that were examined, CR-39^TM^ and polycarbonate, gave similar results, but for simplicity, we used MR-8^TM^ as a model system. (1) First, we performed an ALD passivation process on the SiO_2_/MR-8^TM^ polymer substrate to passivate the polymer substrate and prevent undesired interactions in the SIS stage. (2) We self-assembled polystyrene-*block*-poly(methyl methacrylate) (PS-*b*-PMMA) thin films on the passivated SiO_2_/MR-8^TM^ polymer substrates using a room-temperature process, which includes random copolymer mat UV crosslinking to neutralize the interaction between the SiO_2_ surface and the BCP layer [44], followed by BCP solvent vapor annealing (SVA) to promote BCP self-assembly. (3) We selectively grew AlO_x_ and ZnO in the cylindrical PMMA domains with the AlO_x_/ZnO SIS process. Following SIS, we removed the polymer template with oxygen plasma to obtain AlO_x_/ZnO composite nanorods that can be used as a hard mask for deep reactive ion etching (RIE). (4) We performed an RIE process to break through the Al_2_O_3_ passivation layer and transfer the AlO_x_/ZnO nanorod hard masks into the underlying SiO_2_ layer. The main steps of this process on non-sensitive substrates, SiO_2_/Si and SiO_2_/glass, are presented in Figures S1 and S2.

### 3.2. Room-Temperature BCP Assembly

#### 3.2.1. Random Copolymer Mat Crosslinking Using UV Treatment

Oxide surfaces, such as SiO_2_ and Al_2_O_3_, are favorably wetted by the polar domains of BCP [45]. To induce vertical-oriented assembly, random copolymer mats are commonly used to create a surface that is wetted by both blocks [46]. The required polymer mat layer crosslinking is typically performed with thermal annealing at elevated temperatures (190 °C–250 °C). For example, Figure S3 shows the highly ordered perpendicular orientated self-assembly of PS-*b*-PMMA 46.1k-*b*-21k on a thermally (230 °C) crosslinked PG-4 76% mat. To create a room-temperature BCP assembly process, we first probed room-temperature polymer mat crosslinking using UV exposure (λ = 254 nm) in an inert environment. PG-4 mats are known to be UV crosslinkable due to the presence of epoxy side groups [47].

**Figure 2:**
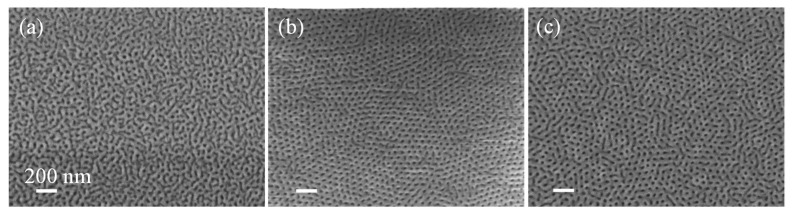
Random copolymer mat crosslinking on SiO_2_/MR-8 polymer substrates. SEM images of PS-b-PMMA self-assembled film (15 min THF SVA) on a PG-4 76% mat layer: (**a**) without UV treatment, (**b**) with 20 secs of UV exposure of a 120 mJ/Cm^2^ dose, and (**c**) with 1 h of UV exposure of a 27,108 mJ/Cm^2^ dose.

The mat layer quality of SiO_2_/Si using the self-assembly of an ~80 nm thick PS-*b*-PMMA 140k-*b*-65k film treated with 15 min of tetrahydrofuran (THF) SVA was examined. Figure 2 presents the resulting PS-*b*-PMMA morphology as a function of UV treatment. The dark domains correspond to PMMA, while the bright domains correspond to PS. When the random copolymer mat, PG-4 76%, was not treated with UV, the resulting PS-*b*-PMMA assembly lacked long-range order (Figure 2a). Applying a short, 20 sec UV treatment of a 120 mJ/cm^2^ dose led to the BCP self-assembly of perpendicular-orientated PMMA cylinders with 42 ± 4 nm diameters and an average grain size of 202 ± 6 nm (Figure 2b). Extensive UV treatment (1 h of exposure with a dose of 27,100 mJ/cm^2^; Figure 2c), resulted in perpendicular assembly but with a lower degree of long-range order.

#### 3.2.2. BCP Solvent Annealing

**Figure 3:**
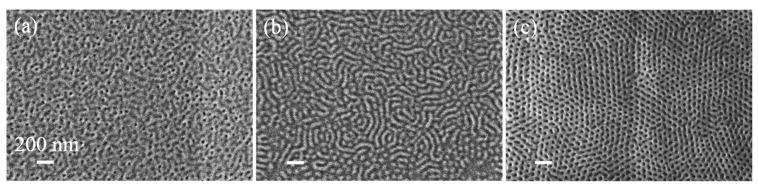
SVA of PS-*b*-PMMA 140k-b-65k on PG4 76%/SiO_2_/Si substrates. SEM images after 15 min of BCP SVA using: (**a**) chloroform, (**b**) acetone, and (**c**) THF.

In order to establish a room-temperature BCP assembly process, we optimized the SVA process for PS-*b*-PMMA 140k-*b*-65k. Vapor infiltration into the polymer film leads to higher chain mobility due to polymer plasticization. It lowers the glass transition temperatures (*T_g_*) and the effective Flory–Huggins parameter of the blocks (χ*_eff_* < χ), as well as creating interface interaction screening, resulting in room-temperature BCP assembly [46,48,49,50,51]. Figure 3 shows the surface morphology of ~80 nm thick PS-*b*-PMMA 140k-*b*-65k after 15 min of SVA using chloroform, acetone, and THF. While chloroform and acetone SVA resulted in disordered assembly and parallel cylinder assembly, respectively (Figure 3a,b), THF SVA resulted in perpendicular PMMA cylinder assembly with 71 ± 2 nm periodicity and a high degree of order. THF is considered a good solvent for both blocks [49,52], swelling both blocks and creating similar effective interfacial energies that lead to perpendicular assembly. Interestingly, when the THF SVA is performed for longer or shorter times than 15 min, the assembly becomes disordered (Figure S4). This “sweet spot” time interval indicates that short SVA might be insufficient to swell both blocks, while long SVA could lead to non-uniform surface interactions due to the differences in polymer block/solvent interactions (χ_PS-THF_ = 0.32 and χ_PMMA-THF_ = 0.8) [53].

#### 3.2.3. SIS on BCP/SiO_2_/MR-8 Polymer

**Figure 4:**
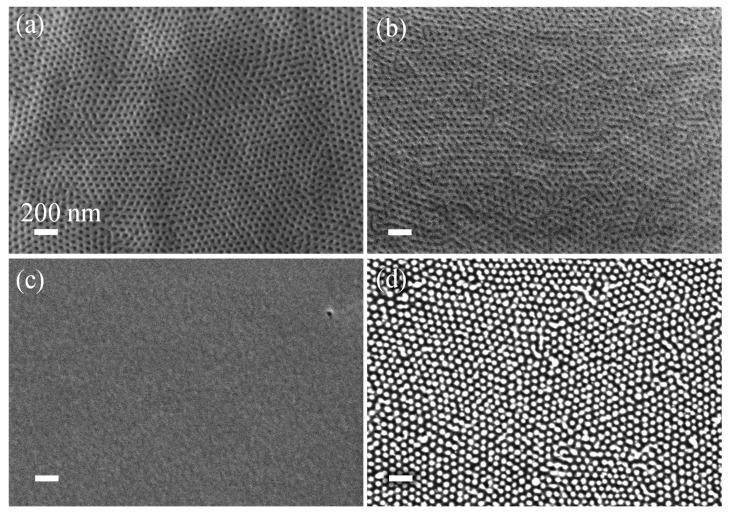
SEM images of PS-*b*-PMMA over SiO_2_/MR-8 substrate: (**a**) self-assembled, (**b**) after 3 h of heat treatment at 80 °C, and (**c**) after eight cycles of AlO_x_ SIS. (**d**) SEM image of AlO_x_ nanocylinders on SiO_2_/Si substrate fabricated using the same process as (**c**).

In order to develop oxide nano-textured surfaces on polymeric substrates, we examined the SIS process on BCP/SiO_2_/MR-8^TM^ polymer stacks. A 500 nm SiO_2_ layer was deposited on an MR-8^TM^ substrate with physical vapor deposition. The prepared substrates (SiO_2_/MR-8^TM^) were then coated with PG4 76% mat and PS-*b*-PMMA 140k-*b*-65k layers, as described in Section 2. BCP assembly on the top of SiO_2_/MR-8^TM^ substrates displayed hexagonally ordered perpendicular PMMA cylinders in a PS matrix (Figure 4a). The thermal stability of the BCP layer was confirmed by placing BCP/SiO_2_/MR-8 samples at 80 °C for 3 h in the ALD chamber without any reaction (Figure 4b). The retention of the BCP self-assembled structure and the absence of SiO_2_/MR-8 polymer degradation during thermal treatment make the PS-*b*-PMMA/SiO_2_/MR-8 stack compatible for the SIS process. However, when we performed between one and eight cycles of AlO_x_ SIS on the PS-*b*-PMMA/SiO_2_/MR-8^TM^ stack, no traces of the self-assembled BCP layer nor a hybrid AlO_x_-BCP layer (Figure 4c) could be identified on the surface.

In comparison, when the same SIS process on PS-*b*-PMMA/SiO_2_/Si and PS-*b*-PMMA/SiO_2_/glass stacks was performed without the presence of the MR-8^TM^ polymer substrate, selective growth of AlO_x_ within the PMMA cylinders could easily be observed. Subsequent etching of the BCP film on SiO_2_/Si substrate with O_2_ plasma resulted in an ordered AlO_x_ nanocylinders array, templated by the PMMA domains, with 42 ± 4 nm diameter AlO_x_ cylinders (Figure 4d and Figure S1). Moreover, when these nanocylinder arrays were used as hard masks for RIE of the underlying SiO_2_ layer on Si or glass substrates, textured SiO_2_ surfaces with tunable-aspect-ratio SiO_2_ nanorods were obtained (Figures S2 and S5). The vertical AlO_x_ nanocylinders and the resulting SiO_2_ nanorods indicate that the PMMA is vertically assembled in the BCP layer and that the SIS process efficiently grew AlO_x_ in the PMMA domains.

While SIS on BCP/SiO_2_/Si and on BCP/SiO_2_/glass substrates resulted in selective growth of AlO_x_ within the PMMA domains, SIS processes on the BCP/SiO_2_/MR-8 polymer substrate did not yield any ordered structure on the top surface. Other polymer substrates that were examined, CR-39^TM^, and polycarbonate, also exhibited the same behavior. We attribute this phenomenon to interactions of the organometallic precursor, TMA, with reactive moieties in the polymer substrate. For example, MR-8^TM^ polymer has an abundance of reactive groups, such as hydroxyl and cyano groups, which can react with TMA and release byproduct molecules. These molecules can partly be deposited on the top BCP layer, obscuring the self-assembled layer and hindering SIS growth within the PMMA domains. SEM characterization of the post-SIS surface and scratching tests (data not shown) indicated the presence of additional organic materials at the top surface.

**Figure 5:**
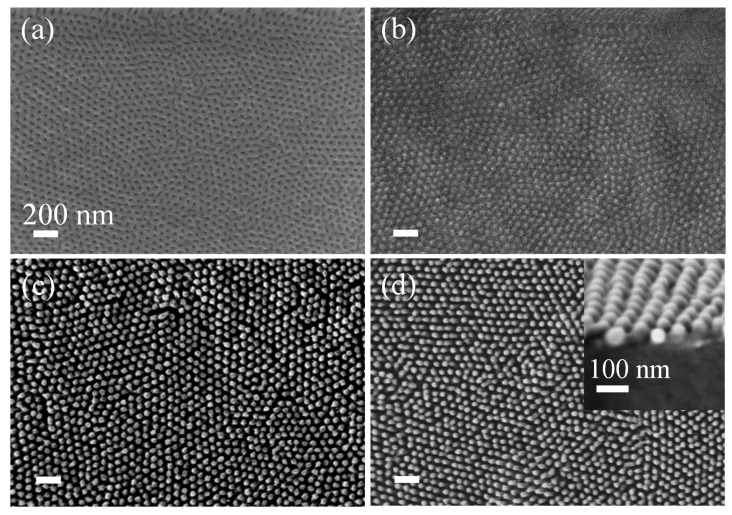
SEM images of (**a**) PS-*b*-PMMA BCP layer assembled over passivated SiO_2_/ MR-8 substrate, (**b**) BCP layer after eight cycles of AlO_x_ SIS, (**c**) AlO_x_ nanocylinders after O_2_ plasma, and (**d**) AlO_x_/ZnO composite cylinders, templated by the layer at (**a**) using two cycles of AlO_x_ SIS and six cycles of ZnO SIS, followed by O_2_ plasma. The inset shows a cross-sectional view of the AlO_x_/ZnO cylinders.

To overcome this limitation and enable SIS-based patterning on polymer substrates, we performed a surface passivation process. The SiO_2_/MR-8^TM^ substrate, prior to random copolymer mat and BCP deposition, was exposed to 100 cycles of Al_2_O_3_ ALD (80 °C, TMA/purge/H_2_O/purge = 10s/10s/10s/10s), resulting in Al_2_O_3_ deposition on the SiO_2_/MR-8^TM^ surface (see illustration in Figure 1). Figure 5 displays SEM imaging of PS-*b*-PMMA 140k-*b*-65k self-assembly and SIS processes on passivated SiO_2_/MR-8^TM^ substrates. Following passivation, we self-assembled PS-*b*-PMMA 140k-*b*-65k on the crosslinked random copolymer mat, as described in Section 2, resulting in highly ordered perpendicular cylinder morphology (Figure 5a). Eight cycles of AlO_x_ SIS on the self-assembled layer resulted in selective growth of AlO_x_ in the PMMA domains, as can be seen from the reverse in contrast to the BCP domains (Figure 5b). We also examined the AlO_x_/ZnO SIS process, where two cycles of AlO_x_ SIS were performed to promote ZnO growth in the PMMA domains using six cycles of ZnO SIS. When we removed the BCP layer using oxygen plasma, we obtained highly ordered AlO_x_ and AlO_x_/ZnO nanorod arrays on the passivated SiO_2_/MR-8^TM^ substrates (Figure 5c and Figure 5d, respectively). A cross-sectional SEM image of AlO_x_/ZnO nanorod arrays is presented as an inset of Figure 5d. The nanorods are estimated to be 25–35 nm in height and 35–40 nm in width. These BCP-templated metal oxide nanorods can now be used as a hard mask for pattern transfer to the underlying SiO_2_ layer.

#### 3.2.4. Pattern Transfer into SiO_2_ Underlying Layer

In order to transfer the BCP-templated metal oxide structure into the SiO_2_ layer, we performed CHF_3_ RIE, which is known for its anisotropic etch of SiO_2_ and good Al_2_O_3_/SiO_2_ selectivity [54,55]. However, due to the SiO_2_/MR-8^TM^ passivation, there was a nanometric Al_2_O_3_ layer between the metal oxide nanorods that needed to be etched to reach the SiO_2_ layer. When we performed CHF_3_ RIE on AlO_x_ nanorods/Al_2_O_3_/SiO_2_/MR-8^TM^, the etch budget in the AlO_x_ nanorods was insufficient to enable Al_2_O_3_ passivation breakthrough and maintain an efficient hard mask for SiO_2_ etching. On the other hand, when we performed CHF_3_ RIE on AlO_x_/ZnO composite nanorods/Al_2_O_3_/SiO_2_/MR-8^TM^, the higher etch resistance of the AlO_x_/ZnO composite nanorods enabled the nanorod to maintain its structure through the Al_2_O_3_ passivation breakthrough etch, leading to efficient pattern transfer.

**Figure 6:**
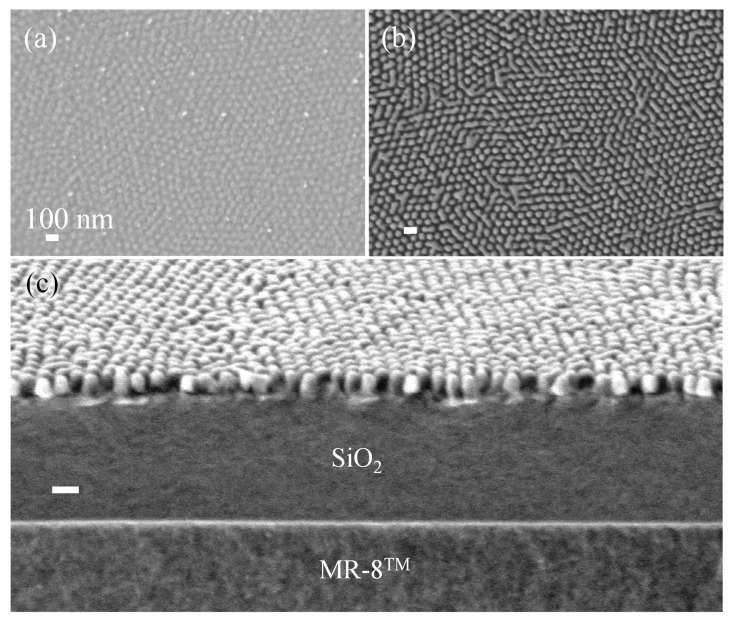
SEM images of AlO_x_/ZnO nanocylinders over an SiO_2_/MR-8 polymer substrate after (**a**) continuous 3 min of CHF_3_ RIE, (**b**) 14 min of loop CHF_3_ RIE. (**c**) Cross-sectional view of a textured SiO_2_ surface generated with the process shown in (**b**). Scale bar is 100 nm in all images.

We further developed the RIE process to account for the low thermal conductivity of the polymer substrate. Figure 6a,b present SEM images of the SiO_2_ surface formed after exposing the BCP-templated AlO_x_/ZnO nanorods to two modes of CHF_3_ RIE: continuous RIE for 3 min (Figure 6a), and loop RIE for 14 min, which is built from loops of 30 s of RIE and a 1 min purge (Figure 6b). The continuous 3 min etching resulted in shallow SiO_2_ texturing, as implied by the low contrast of the structure. The low thermal conductivity of the polymer substrate surface leads to substrate heating, enhancing isotropic etching, which results in nanocylinder mask etching. The loop RIE process, on the other hand, provides sufficient time between the plasma periods to dissipate the heat generated on the surface. Figure 6b clearly shows the intactness of the hexagonally ordered cylindrical array formed after the loop RIE. The corresponding cross-sectional image (Figure 6c) shows ~80 nm high and ~40 nm wide (~2:1 aspect ratio) oval-shaped nanorods, fabricated in the SiO_2_ layer by room-temperature BCP self-assembly, AlO_x_/ZnO SIS, and loop RIE. Further optimization of RIE chemistry for deep and/or conical-shaped high-aspect-ratio nanostructures is viable using the methodology presented in Figure S5.

## 4. Conclusions

In this research, a new methodology for creating highly ordered and periodic, nanoscale oxide texture on top of polymeric substrates was developed. These nanoscale textures can be used to enhance various properties, such as anti-reflectiveness and superhydrophobicity. The texturing is based on transferring the pattern of BCP onto metal oxide nanorods via SIS and onto the oxide layer via RIE. Our approach provided solutions to the challenges that nanoscale oxide texturing of polymer substrates poses. We have developed a series of suitable low-temperature (<80 °C) processes geared towards temperature-sensitive substrates. The polymer substrates were 3D-passivated with an ALD-processed Al_2_O_3_ barrier layer. On top of preventing undesired interactions between the SIS organometallic precursors and the polymer substrates, the passivation layer can be used as a general basis layer, reducing the need to tune the process for each polymer substrate chemistry. In addition, in order to enable a differential etch rate in the RIE step, an AlO_x_/ZnO composite was generated in the PMMA cylinder phase. This combined barrier-differential etch approach expands the standard SIS toolbox to enable new pathways for nanofabrication on a wide array of lighter-weight polymers over traditional SIS-processed glass and silicon substrates.

## Data Availability

Data available in a publicly accessible repository.

## Supplementary Materials

The following are available online at https://www.mdpi.com/article/10.3390/polym13132209/s1. Figure S1: SEM images of AlO_x_ cylinders pattern formed on (a) SiO_2_/Si substrate and (b) SiO_2_/glass substrate; Figure S2: Top (a,c) and cross-sectional (b,d) SEM images of SiO_2_ nanostructures formed by etching the AlO_x_ nanocylinder pattern on SiO_2_/Si substrate (a,b) and SiO_2_/glass substrate (c,d) using 10 min CHF_3_ RIE; Figure S3: Top view SEM image of (a) PS-*b*-PMMA (46.1k-*b*-21k) block/copolymer after thermal annealing at 230 °C; Figure S4: SEM image of PS-*b*-PMMA (140k-*b*-65k) over SiO_2_/Si substrate vapor annealed for 10 and 20 min in tetrahydrofuran; Figure S5: SEM image of SiO_2_ nanorods array fabricated by different RIE chemistry: anisotropic etching of SiO2 by CHF_3_ (a) 5 min, (b) 10 min; isotropic etching of SiO_2_ by CF_4_ + O_2_ (c) 3 min, (d) 4 min, and combination of CHF_3_ and CF_4_ +O_2_ (e) 5 min and 1 min, (d) 10 min and 0.5 min, respectively.
