# Supplementary material for: Fabrication of Nanoscale Oxide Textured Surfaces on Polymers
Source: Polymers (Basel). 2021 Jul 3;13(13):2209. doi: 10.3390/polym13132209 (PMC8271387; doi:10.3390/polym13132209)
Supplement: Supplementary file 1 [file polymers-13-02209-s001.zip › polymers-1281096-supplementary.pdf]

Supplementary information

Nanoscale texturing of oxide layers on polymers

Barun K. Barick[1], Neta Shomrat[1], Uri Green[2], Zohar Katzman[2], Tamar Segal-Peretz[1*]

[1]Department of Chemical Engineering, Technion, Haifa-3200003, Israel
[2]Shamir Optical Industry Ltd, Israel
Corresponding author's email: tamarps@technion.ac.il

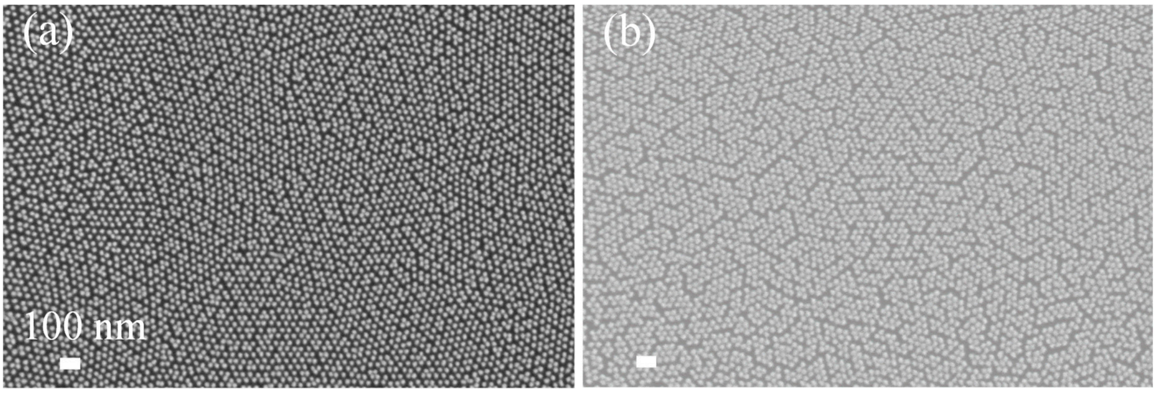

Figure S1. SEM images of AlO_x cylinders pattern formed on: (a) SiO_2/Si substrate and (b) SiO_2/glass substrate. The AlO_x cylinders were fabricated using thermally annealed PS-*b*-PMMA 46.1k-*b*-21k which were subjected to eight cycles of AlO_x SIS and subsequent O_2 etching.

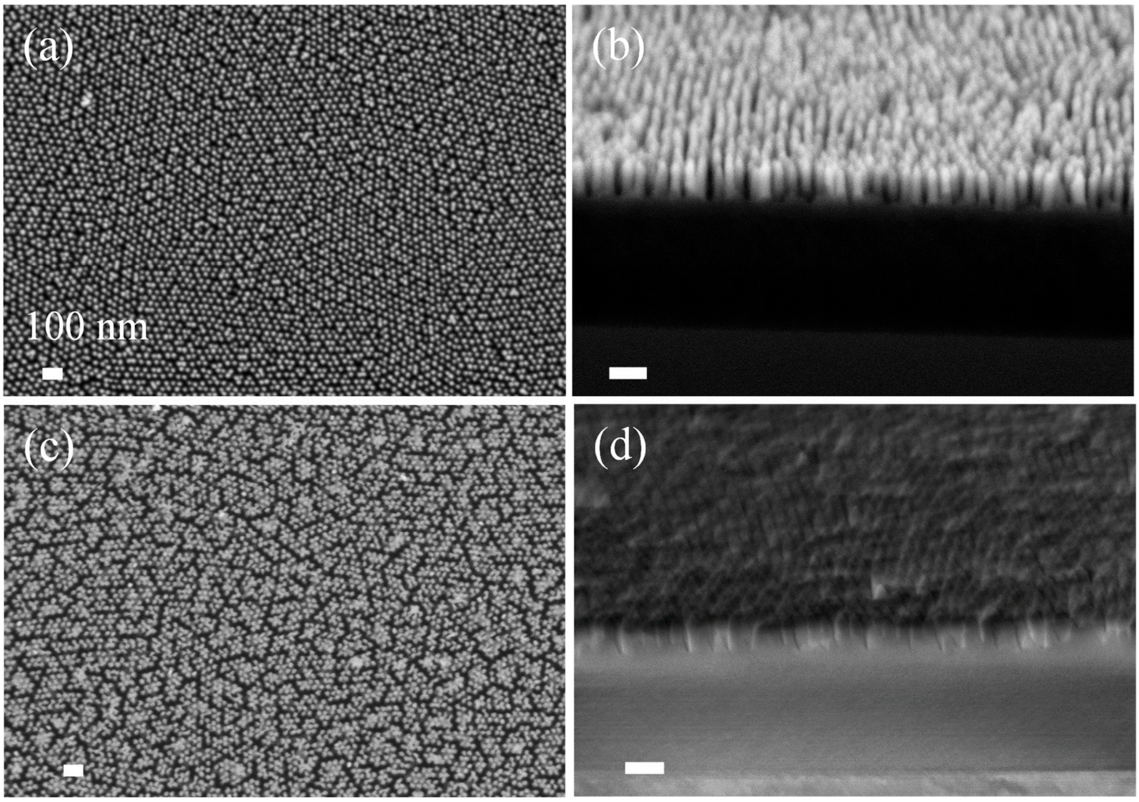

Figure S2. Top (a, c) and cross-sectional (b,d) SEM images of SiO_2 nanostructures formed by etching the AlO_x nanocylinder pattern on SiO_2/Si substrate (a,b) and SiO_2/glass substrate (c, d) using 10 min CHF_3 RIE. The average height of nanorods is found to be 104 ± 2 nm.

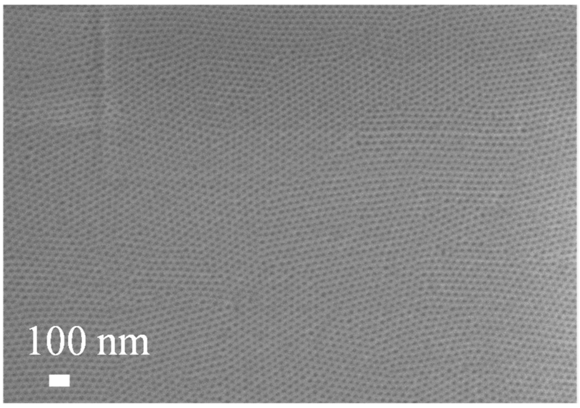

Figure S3. Top view SEM image of (a) PS-*b*-PMMA (46.1k-*b*-21k) block-copolymer after thermal annealing at 230 °C. Perpendicular domain orientation of PMMA in the copolymer layer is observed by neutralization of the surface with a P(S-r-MMA-r-GMA) random copolymer mat layer thermal annealed at 250 °C.

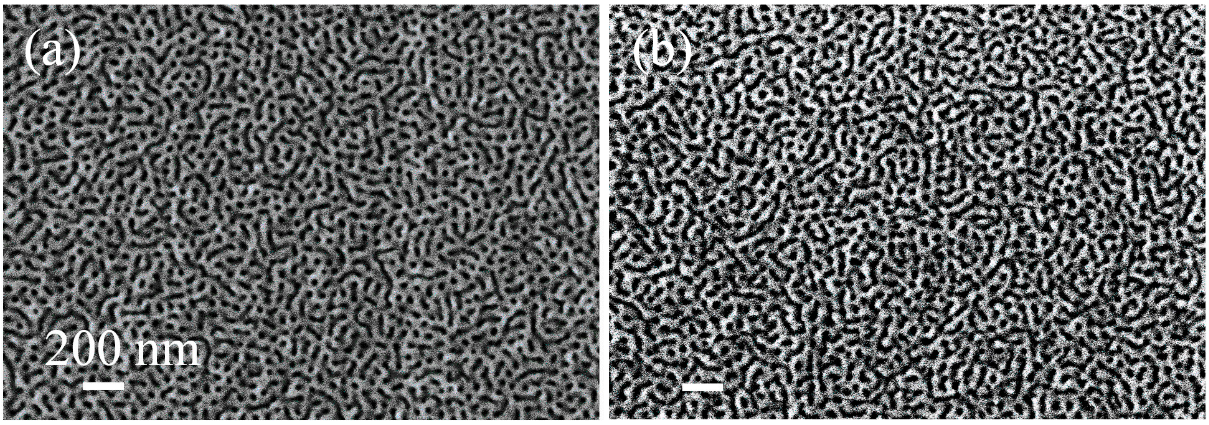

Figure S4. SEM image of PS-b-PMMA (140k-b-65k) over SiO_2/Si substrate vapor annealed for 10 and 20 min in tetrahydrofuran. Prior to BCP, the substrate was neutralized by 0.3 wt% PG-4 77%PS mat layer (UV in optimal crosslink).

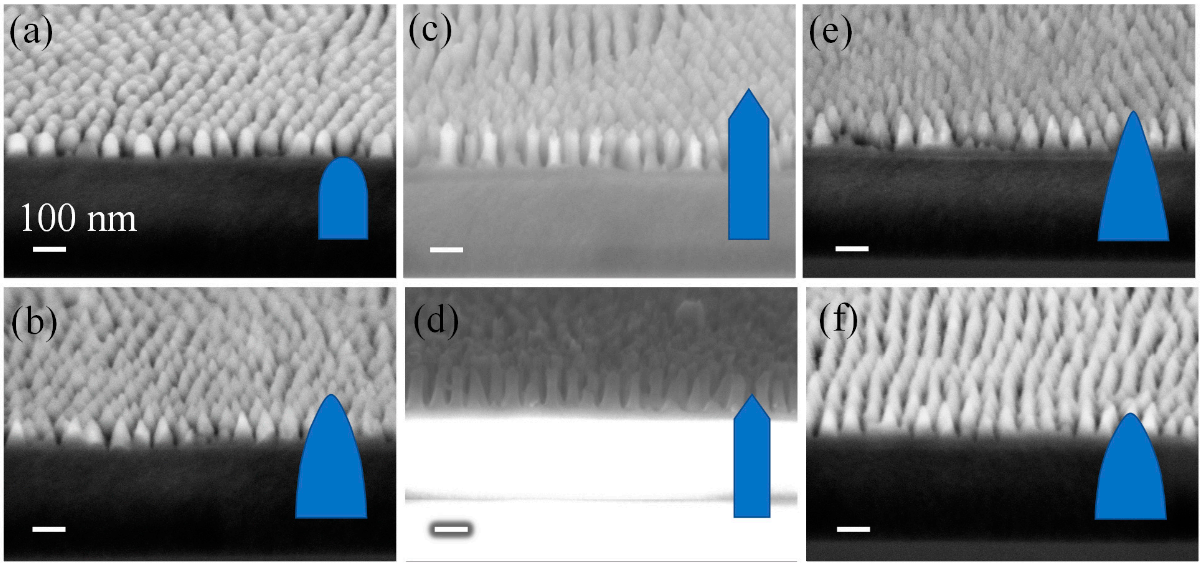

Figure S5. SEM image of SiO_2 nanorods array fabricated by different RIE chemistry: anisotropic etching of SiO_2 by CHF_3 (a) 5 min (b) 10 min; isotropic etching of SiO_2 by CF_4+O_2 (c) 3 min, (d) 4 min, and combination of CHF_3 and CF_4 +O_2 (e) 5 min and 1 min (d) 10 min and 0.5 min, respectively. Respective, inset shows schematic of nanorod morphology (mentioned height and width in nm).

The morphology of nanostructures plays a vital role in controlling the antireflective property of a surface. To pattern different morphologies, isotropic and anisotropic based RIE chemistry are employed to etch nanocylinder patterned SiO_2/Si substrate. Figure S5 shows the SEM image of SiO_2 nanorods array fabricated by different RIE chemistry, and corresponding schematic morphology is shown in the respective inset. Figures S5a and S5b are the SEM image recorded after 5 and 10 min of RIE using CHF_3. Anisotropic etching of SiO_2 leads to oval-shaped cylinders. Height and base width are measured to be 87±5 and 51±3 nm for 5 min of RIE. Moreover, more extended etching of 10 min RIE facilitates side etch, creating paraboloidal nanostructures with 103±4 nm of height and 56±2 nm of the base width. Figures S5c and S5d are the SEM image recorded after 3 and 4 min of RIE using isotropic etching of SiO_2 by CH_4+O_2. Cylindrical nanorods are observed with CH_4+O_2 RIE due to enhanced side etching as oxygen reduces the deposition of fluorocarbon inhibitor on sidewall. Height and width are found to be 128±5 and 38±2 nm for 3 min of RIE, which is reduced to 111±4 and 32±4 nm for 4 min case indicating the verge of AlO_x nanocylinder mask etching. For broadband antireflective coating, a high aspect ratio conical-shaped nanorod with base connected to each other is desired. A successive anisotropic and isotropic etching, i.e., RIE by CHF_3, followed by CF_4 +O_2, produces conical structures for 5 min and 1 min, respectively (Figure S5e) and paraboloid for 10 min and 0.5 min, respectively (Figure S5f). Height and base width of cones are found to be 112±4 and 55±7 nm for the former case, due to etching from top, height reduce to 79±6 nm with the base width of 58±3 nm for the latter case.
